# The palmitoylation code in cell death signaling: from molecular mechanisms to therapeutic opportunities

**DOI:** 10.3389/fcell.2026.1740922

**Published:** 2026-02-24

**Authors:** Han-xi Xiao, Zhou-zhou Li, Xu-huan Li, Ting Chen, Xin-rong Tang, Yu Zhang, Zu-xiu Wang, Yong-ping Pan

**Affiliations:** 1 Affiliated Rehabilitation Hospital, Jiangxi Medical College, Nanchang University, Nanchang, Jiangxi, China; 2 Rehabilitation College, Jiangxi Medical College, Nanchang University, Nanchang, Jiangxi, China; 3 Tongde Hospital of Zhejiang Province Affiliated to Zhejiang Chinese Medical University, Hangzhou, Zhejiang, China; 4 Jiangsu Engineering Research Center of Medical Genetics and Transformation, Department of Genetics, Xuzhou Medical University, Xuzhou, Jiangsu, China

**Keywords:** apoptosis, cancer, cell death, ferroptosis, neurodegenerative disease, palmitoylation, programmed cell death, pyroptosis

## Abstract

The dynamic lipid modification known as protein palmitoylation is essential for modulating protein activity and subcellular distribution. This process is increasingly recognized as a pivotal molecular mechanism governing the balance between cellular survival and death. This paper explores the molecular regulation of palmitoylation within diverse pathways of regulated cell death, for instance, in necroptosis, ferroptosis, pyroptosis, and apoptosis. The core findings indicate that by controlling the stability, membrane anchoring, and interactions of key signaling proteins, palmitoylation can precisely regulate the ultimate fate of the cell. Additionally, the dysregulation of palmitoylation is closely linked to the pathogenesis of major human diseases, including cancer, neurodegenerative disorders, and inflammatory diseases. For instance, the process can display a dual role in tumor progression, acting to either promote or inhibit it. Concurrently, it is essential for the inflammatory signaling in pyroptosis and for mounting a cellular defense against ferroptosis. A deeper understanding of these regulatory networks provides highly promising therapeutic targets for disease intervention. Targeting the activity of specific palmitoylation-related enzymes has emerged as an innovative strategy for developing novel therapies for a range of diseases, demonstrating significant clinical translational potential.

## 1 Introduction

Protein palmitoylation is a form of dynamic lipidation that occurs after protein synthesis. This reversible process is defined by the covalent attachment of palmitic acid, a 16-carbon fatty acid, to the thiol groups of cysteine amino acids in a polypeptide chain. This form of lipidation is of central importance to a multitude of normal and disease-related cellular functions. Its significance stems from its capacity to fine-tune protein stability, subcellular trafficking, and the formation of molecular complexes (Mesquita et al., 2024). Among all types of palmitoylation, S-palmitoylation is the most prevalent and extensively studied. This process forms a thioester linkage between palmitate and cysteine thiols, and the reversible nature of this bond provides a mechanism for the precise spatiotemporal control of protein activity. In contrast, N-palmitoylation and O-palmitoylation are less studied in the context of dynamic cell signaling because the chemical bonds they form are more stable and generally irreversible (Hornemann, 2015).

The dynamic equilibrium of palmitoylation is precisely regulated by two key classes of enzymes: palmitoyl acyltransferases (PATs) and acyl-protein thioesterases (APTs). PATs belong to a protein family characterized by a zinc-finger domain (Asp-His-His-Cys, DHHC) and are responsible for catalyzing the attachment of palmitic acid onto target proteins. Twenty-three different ZDHHC enzymes have been identified in the human genome (Rana et al., 2018; Mesquita et al., 2024). The expression of ZDHHC enzymes in the human body exhibits significant heterogeneity and high tissue specificity. The brain is the organ with the most active S-palmitoylation modification (Liao et al., 2024). Notably, ZDHHC5 is highly expressed in excitatory neurons of the hippocampus and cortex, where it participates in the formation of learning and memory (Liao et al., 2024), whereas ZDHHC2 is widely expressed in cortical neurons and is crucial for maintaining synaptic structural stability (Wild et al., 2022). In the immune system, ZDHHC enzymes control the threshold and intensity of immune responses by regulating the membrane assembly of signaling complexes (Chen et al., 2025). For instance, in T cells, ZDHHC6 and ZDHHC18 are involved in regulating the stability of immune checkpoint proteins, thereby maintaining immune homeostasis (Tang et al., 2024). In metabolically active organs such as the liver, pancreas, and heart, the maintenance of tissue function also relies on the regulation of substrate uptake and ion balance by ZDHHC enzymes (Baldwin et al., 2023; Liu et al., 2025). It is noteworthy that the activity of ZDHHC enzymes is not constant but is precisely regulated by transcriptional levels, post-translational modifications, accessory protein networks, and small molecule metabolites. For example, ZDHHC5 is dynamically regulated by phosphorylation (Liao et al., 2024); Golgin A7 (GOLGA7) and its paralog GOLGA7B serve as key activators for ZDHHC9 and ZDHHC5, respectively (Salaun et al., 2020); while 2-bromopalmitate (2-BP) acts as a broad-spectrum inhibitor of ZDHHC enzymes (Lan et al., 2021). Furthermore, the catalytic mechanism of ZDHHC enzymes relies on a nucleophilic cysteine thiolate anion, a chemical group that is extremely sensitive to oxidative modifications. Typically, reactive oxygen species (ROS) can directly oxidize the cysteine to form sulfenic acid (-SOH). If oxidation persists, it may further form disulfide bonds with glutathione or adjacent cysteines, or even convert into irreversible sulfinic/sulfonic acids (-SO_2_H/-SO_3_H), leading to a global reduction in protein palmitoylation levels (Paulsen and Carroll, 2010; Hong et al., 2024). However, in pathological models of pressure-overloaded hearts, ROS can activate the expression of ZDHHC3/7 via transcription factors. High levels of ZDHHC3/7 lead to the hyper-palmitoylation of Ras-related C3 botulinum toxin substrate 1 (Rac1), promoting its translocation to the plasma membrane to assemble and activate the Nicotinamide Adenine Dinucleotide Phosphate (NADPH) oxidase complex, which subsequently generates more ROS, thereby creating a vicious cycle (Baldwin et al., 2023).

**FIGURE 1:**
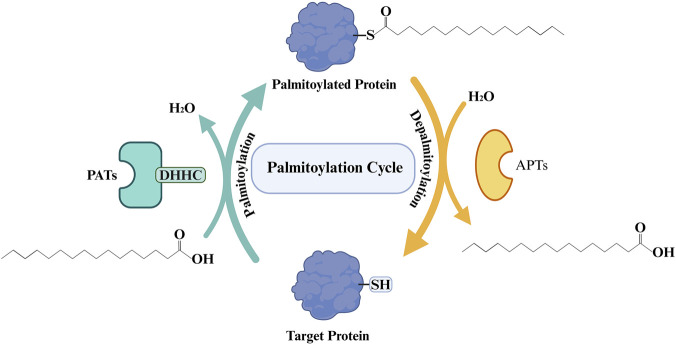
Palmitoylation Cycle. DHHC: Asp-His-His-Cys; PATs: palmitoyl acyltransferases; APTs: acyl-protein thioesterases.

Conversely, the removal of palmitate is catalyzed by several thioesterases, including Acyl-protein Thioesterase 1/2 (APT1/2), Palmitoyl-protein Thioesterase 1/2 (PPT1/2), and the Abhydrolase Domain Containing 17 (ABHD17) family, which hydrolyze the thioester linkage to detach the fatty acid (Mesquita et al., 2024). The interplay between PATs and these depalmitoylating enzymes establishes the complete “palmitoylation cycle,” enabling the transient modulation of protein activity (Figure 1).

The process of cellular death is a core biological phenomenon crucial for maintaining organismal equilibrium, guiding normal growth, and remodeling tissues. The classification of cell death modalities is primarily defined by specific molecular mechanisms rather than morphological features. A wide spectrum of Regulated Cell Death (RCD) routines falls under this classification, encompassing Apoptosis, Pyroptosis, Necroptosis, and Autophagy-Dependent Cell Death. Furthermore, the classification distinguishes these from biologically uncontrolled Accidental Cell Death (ACD), while incorporating other specific RCD modalities such as Ferroptosis, Parthanatos, Lysosome-Dependent Cell Death, and NETotic Cell Death (Galluzzi et al., 2018).

Considering the fundamental role that palmitoylation plays in directing protein function, its specific regulatory effects on cell death signaling are now a burgeoning field of study. As a widespread dynamic modification, palmitoylation can directly or indirectly influence key regulatory proteins involved in various cell death pathways, thereby determining the ultimate fate of the cell (Table 1). Accumulating research indicates that clarifying the molecular mechanisms of palmitoylation’s role in cellular demise can significantly advance our knowledge of core biological principles. Moreover, this line of inquiry is uncovering valuable therapeutic targets for designing novel treatments against conditions such as cancer, neurodegenerative disorders, and infectious or inflammatory diseases (Lin et al., 2023; Wlodarczyk et al., 2024; Lu et al., 2025).

**TABLE 1:** Palmitoylation in regulating cell death.

Type of cell death	Key associated protein	Palmitoylation site (s)	Role of palmitoylation	Palmitoyl acyltransferases or acyl-protein thioesterases
Apoptosis	BAX	Cys126	Palmitoylation promotes the mitochondrial localization and oligomerization of key effector proteins, a process which in turn facilitates caspase activation and the formation of the apoptosome (Fröhlich et al., 2014)	ZDHHC family
	RIPK1	Cys256 (human) Cys257 (murine)	By increasing the hydrophobicity of the RIPK1 kinase domain, palmitoylation promotes its homotypic interactions, which enhances the trans-autoactivation of RIPK1 and ultimately initiates downstream cell death signaling pathways (Zhang et al., 2024c)	ZDHHC5
Autophagy-dependent cell death	ULK1	Cys927 (yeast); Cys1003 (yeast)	Palmitoylation enhances the hydrophobic interaction between ULK1 and phospholipids, which in turn facilitates the recruitment of downstream complexes (Tabata et al., 2024)	ZDHHC13
	Beclin 1	Cys137	Palmitoylation enhances the stability of the core autophagy complex, PI3K (Guo et al., 2024)	ZDHHC5
	P62	Cys289 (human); Cys290 (human)	Palmitoylation enhances the affinity of p62 for the LC3-positive autophagosome membrane, thereby promoting the formation of p62 droplets containing ubiquitinated substrates and their subsequent engulfment by the autophagosome (Huang et al., 2023)	ZDHHC19
	P62	Cys289 (human); Cys290 (human)	Depalmitoylation leads to an increase in p62 lipid droplet size and a decreased association with LC3, thereby significantly reducing the efficiency of selective autophagy (Huang et al., 2023)	APT1
	NOD2	Cys395 (human); Cys1033 (human)	Palmitoylation inhibits the SQSTM1/p62-dependent autophagic degradation of NOD2, thereby enhancing its stability. This subsequently triggers the downstream NF-κB pathway, leading to an associated inflammatory reaction (Zhou et al., 2022)	ZDHHC5
	ATG9A	Cys155 (human); Cys156 (human)	Palmitoylation promotes the binding of ATG9 to the AP-4 complex and its subsequent trafficking from the Golgi apparatus to the phagophore membrane, thereby ensuring the efficiency of autophagosome initiation and elongation (Xia et al., 2025)	ZDHHC5
	Ykt6	Cys183 (human); Cys186 (human)	Palmitoylation enables Ykt6 to form a priming complex with STX17 and SNAP29, which facilitates the subsequent recruitment of VAMP8 to drive autolysosome formation (Zheng et al., 2024)	ZDHHC family
	Oct4A	Cys198 (human)	Palmitoylation maintains the stemness and tumorigenicity of GSCs (Chen et al., 2023)	ZDHHC17
Necroptosis	RIPK1	Cys256 (human) Cys257 (murine)	By increasing the hydrophobicity of the RIPK1 kinase domain, palmitoylation promotes its homotypic interactions, which enhances the trans-autoactivation of RIPK1 and ultimately initiates downstream cell death signaling pathways (Zhang et al., 2024c)	ZDHHC5
Pyroptosis	NLRP3	Cys130 (human); Cys958 (human); Cys126 (murine); Cys955 (murine)	Palmitoylation promotes the dynamic trafficking of NLRP3 between mitochondria, the TGN, and endosomes, ultimately directing its transport to the MTOC (Nie et al., 2024)	ZDHHC1
	NLRP3	Cys126 (human)	Palmitoylation maintains NLRP3 in a state of low solubility, thereby lowering its threshold for phase separation (Zou et al., 2025)	ZDHHC7
	NLRP3	Cys126 (human)	Depalmitoylation negatively controls the activation of the NLRP3 inflammasome (Schmacke and Hornung, 2025)	ABHD13
	NLRP3	Cys837 (human); Cys838 (human)	The assembly of the inflammasome is promoted by an improved association between NEK7 and the LRR domain of NLRP3, an interaction that is strengthened by palmitoylation (Zheng et al., 2023)	ZDHHC5
	NLRP3	Cys837 (human); Cys838 (human)	Depalmitoylation reverses the corresponding ZDHHC5-mediated palmitoylation (Zheng et al., 2023)	ABHD17A
	NLRP3	Cys419 (human)	Palmitoylation further stabilizes the NACHT domain of NLRP3, thereby accelerating inflammasome assembly (Hu et al., 2024)	ZDHHC17
	NLRP3	Cys841 (human); Cys844 (murine)	Palmitoylation facilitates the autophagic degradation of NLRP3 (Wang et al., 2023)	ZDHHC12
	NLRP3	Cys6 (murine)	Depalmitoylation promotes the degradation of NLRP3 (Lv et al., 2023)	PPT1
	GSDMD	Cys191 (human); Cys192 (murine)	Palmitoylation increases the hydrophobicity of the GSDMD-NT, mediating its recruitment and anchoring to the plasma membrane. Palmoylation assists in the optimal positioning or recognition for Caspase-mediated cleavage. (Zhang et al., 2024c)	ZDHHC7
	GSDMD	Cys191 (human) Cys192 (murine)	Palmitoylation enhances the hydrophobicity of the GSDMD-NT, mediating its recruitment and stable anchoring to the plasma membrane. Without palmitoylation, cleaved GSDMD fails to form pores, preventing pyroptosis (Balasubramanian et al., 2024; Du et al., 2024)	ZDHHC5/9
	GSDMD	Cys191 (human); Cys192 (murine)	Depalmitoylation exposes the cysteine residues necessary for intermolecular interactions, thereby driving pore formation and executing pyroptosis. (Zhang et al., 2024c)	APT2
Ferroptosis	SLC7A11	Cys327 (human)	Palmitoylation inhibits the proteasomal degradation of SLC7A11, thereby enhancing its protein stability (Wang et al., 2024)	ZDHHC8
	GPX4	Cys66 (human)	Palmitoylation enhances protein stability by reducing ubiquitin-mediated degradation (Huang et al., 2025)	ZDHHC20
	GPX4	Cys75	Palmitoylation promotes the anchoring of GPX4 to the endoplasmic reticulum membrane, thereby enhancing the efficiency of its clearance of lipid peroxides (Zhou et al., 2025)	ZDHHC8
	GPX4	Cys66 (human)	Depalmitoylation causes GPX4 to dissociate from the membrane and accelerates its degradation, thereby promoting ferroptosis (Huang et al., 2025)	APT2
Lysosome-Dependent Death	SLC7A11	Cys327 (human)	By inhibiting the ubiquitination and subsequent lysosome-dependent degradation of SLC7A11, palmitoylation enhances cellular resistance to ferroptosis, thereby indirectly regulating lysosome-mediated degradation processes (Wang et al., 2024)	ZDHHC8
	ATG16L1	Cys153 (human)	Palmitoylation enables the binding of ATG16L1 to phosphatidylinositol, a process that enhances autophagosome formation and lysosomal fusion. Under conditions of excessive autophagy, however, this can potentially trigger lysosome-dependent death (Wei et al., 2024)	ZDHHC7
	PD-L1	Cys272 (human)	Palmitoylation prevents the degradation of PD-L1 via the lysosomal pathway, thereby indirectly regulating lysosome-dependent death (Yao et al., 2019)	ZDHHC3

Bax, Bcl-2-associated X protein; RIPK1, Receptor-Interacting Protein Kinase 1; ULK1, Unc-51 Like Autophagy Activating Kinase 1; Beclin 1, Bcl-2-interacting coiled-coil protein 1; PI3K, Phosphoinositide 3-kinase; SQSTM1, It is usually referred to as p62, Sequestosome 1; LC3, 1A/1B-light chain 3; NOD2s, Nucleotide-binding oligomerization domain-containing protein 2s; NF-κB, kappa-light-chain-enhancer of activated B cells; ATG, Autophagy-Related Gene; AP-4, Adaptor Protein 4; Ykt6, Synaptobrevin homolog YKT6; STX17, Syntaxin 17; SNAP29, Synaptosomal-Associated Protein 29; VAMP8, Vesicle-Associated Membrane Protein 8; Oct4A, Octamer-binding transcription factor 4A; GSC, Glioblastoma Stem Cells; NLRP3, NLR family pyrin domain containing 3; TGN, Trans-Golgi Network; MTOC, Microtubule-Organizing Center; LRR, Leucine-Rich Repeat; NEK7, Nima-related kinase 7; GSDMD, Gasdermin D; GSDMD-NT:GSDMD-N terminal fragment; SLC7A11, Solute Carrier Family 7 Member 11; GPX4, Glutathione Peroxidase 4; ATG16L1, Autophagy related 16 like 1; PD-L1, Programmed death-ligand 1; ZDHHC, Zinc finger DHHC-type palmitoyltransferase; APT1, Acyl-protein Thioesterase 1; PPT1, Palmitoyl-protein Thioesterase 1; ABHD17, Abhydrolase Domain Containing 17.

## 2 Palmitoylation as a molecular controller of cell death pathways

### 2.1 Apoptosis

The apoptotic process is primarily initiated via two major routes: an extrinsic pathway triggered by death receptor engagement and an intrinsic pathway centered on the mitochondria. The latter’s crucial event, releases of cytochrome c into the cytosol and dissipation of the membrane potential, which activates a Caspase protease cascade. This entire sequence defines apoptosis, a type of regulated cell death orchestrated by precise genetic program. S-palmitoylation, a dynamic lipid attachment, serves as a pivotal modulator of apoptosis by influencing key protein components within both pathways.

In the intrinsic apoptosis pathway, palmitoylation primarily affects mitochondrial function and signal transduction by modifying key regulatory proteins. Protein palmitoylation can drive the intrinsic apoptotic pathway by targeting key members of the B-cell lymphoma 2 (Bcl-2) family. This modification induces Mitochondrial Outer Membrane Permeabilization (MOMP) through the direct lipidation of pro-apoptotic proteins like B-cell lymphoma 2 (Bcl-2)-associated X protein (Bax) and Bcl-2 homologous antagonist/killer (Bak). The attachment of palmitate thereby enhances their recruitment and assembly at the outer mitochondrial membrane. For instance, in vascular endothelial cells, palmitoylation leads to an upregulation of Bax expression and a decrease in the Bcl-2/Bax ratio, which subsequent to causes the discharge of cytochrome c and the activation of Caspase-3 (Xu et al., 2019).

Further studies have confirmed that the cysteine residue at position 126 (Cys126) on the Bax protein is its primary site for S-palmitoylation (Fröhlich et al., 2014). Palmitoylation at this site is crucial for its mitochondrial targeting, oligomerization capacity, and pro-apoptotic activity. If Cys126 is mutated to a palmitoylation-deficient form, it results in reduced mitochondrial localization of Bax, impaired oligomerization, decreased Caspase-3 activity, and ultimately, the inhibition of apoptosome formation (Fröhlich et al., 2014). In hepatocytes where the Growth Hormone Receptor is silenced, exposure to palmitic acid (PA) induces a notable increase in mitochondrial ROS, along with depolarization and the activation of Caspase-9. Importantly, these effects were efficiently nullified by the application of MitoTEMPO, an antioxidant specifically targeting the mitochondria (Wang et al., 2021). In a model of high-fat-induced hepatocyte apoptosis, the palmitoylated fatty acid translocase Cluster of Differentiation 36 (CD36) enhances the cellular uptake of fatty acids (Zeng et al., 2022). The process culminates in a buildup of mitochondrial ROS while also cooperatively triggering the endoplasmic reticulum stress response. This response then mediates the liberation of cytochrome c via the C/EBP homologous protein (CHOP)/Bcl-2 pathway, resulting in the eventual activation of the Caspase cascade (Wang et al., 2021; Zeng et al., 2022).

In the death receptor-mediated apoptosis route, S-palmitoylation influences subsequent signaling events and the formation of the Death-Inducing Signaling Complex (DISC). This occurs through the modification of pivotal components, a prominent one being Receptor-Interacting Protein Kinase 1 (RIPK1), a key nexus in the Tumor Necrosis Factor (TNF) cascade. Upon TNF stimulation, K63-linked ubiquitinated RIPK1 recruits the palmitoyltransferase DHHC5 to the cell membrane, which then catalyzes the palmitoylation of RIPK1 at its Cys257 site (Zhang N. et al., 2024). This modification creates a hydrophobic microenvironment that significantly enhances the interaction of RIPK1 with apoptotic signal-transducing proteins, exemplified by Fas-Associated Death Domain (FADD) and Caspase-8, which initiate the apoptotic program (Zhang N. et al., 2024). In a model of Metabolic-associated steatohepatitis (MASH), the accumulation of fatty acids in hepatocytes upregulates DHHC5 expression, which in turn enhances RIPK1 palmitoylation and exacerbates hepatocyte apoptosis and inflammatory damage (Zhang N. et al., 2024). In the case of the Fas receptor/Cluster of Differentiation 95 (CD95), a different classic death receptor, the direct lipidation of the receptor itself has yet to be demonstrated, necessitating additional research.

In conclusion, while protein palmitoylation clearly acts as a central regulatory level influencing both intrinsic and extrinsic apoptosis—via control of mitochondrial BCL-2 proteins and DISC assembly—a more detailed overview of how these lipid modifications are coordinated requires further investigation.

### 2.2 Autophagy-dependent cell death

Autophagy-dependent Cell Death is mechanistically reliant on the process of autophagy. In contrast to apoptosis, this form of cellular demise is not contingent on Caspase family activation but is caused by unrestrained autophagic activity. If the level of autophagy surpasses a cell’s homeostatic limits, the widespread degradation of components inflicts irreversible damage on vital organelles and proteins, leading to a breakdown of cellular stability and death (Denton and Kumar, 2019). As a key post-translational modification, palmitoylation profoundly influences every stage of the autophagosome lifecycle—including initiation, elongation, and maturation—by regulating the membrane localization, and function of core proteins in the autophagy pathway (Zhou et al., 2022; Guo et al., 2024).

Palmitoylation plays multiple roles in regulating autophagosome biosynthesis. In the initial stages of autophagy, the ZDHHC13-catalyzed acylation of the Unc-51 Like Autophagy Activating Kinase 1 (ULK1) complex strengthens its hydrophobic connection with the phospholipid bilayer of membranes. This process is essential for attracting the downstream Phosphoinositide 3-kinase (PI3K) complex to the membrane of the endoplasmic reticulum, which in turn triggers phagophore biogenesis (Tabata et al., 2024). Subsequently, during the elongation of the autophagosome membrane, the palmitoylation of Bcl-2-interacting coiled-coil protein 1 (Beclin 1) —a core component of the PI3K complex—mediated by enzymes such as ZDHHC5, promotes its binding to Autophagy Related 14 Like (ATG14L) and Vacuolar Protein Sorting 15 (VPS15) (Guo et al., 2024). This enhances the stability and catalytic activity of the PI3K complex, thereby accelerating autophagosome formation (Guo et al., 2024). In addition to core components, palmitoylation also regulates selective autophagy. Palmitoylation of the autophagy receptor Sequestosome 1 (SQSTM1,It is usually referred to as p62) enhances its affinity for the Microtubule-associated protein 1A/1B-light chain 3 (LC3)-positive autophagosome membrane, thus promoting the formation of p62 droplets containing ubiquitinated substrates and their engulfment by the autophagosome (Huang et al., 2023). Conversely, the process of depalmitoylation has been shown to induce an augmentation in the dimensions of p62 lipid droplets, concomitant with a diminished association with LC3 (Huang et al., 2023). This results in a substantial reduction in the efficacy of selective autophagy (Huang et al., 2023). Furthermore, palmitoylation can link autophagy with other signaling pathways. ZDHHC5-mediated palmitoylation of the Nucleotide-binding oligomerization domain-containing protein 2s (NOD2s) protein inhibits its degradation by autophagy (Zhou et al., 2022). The consequence is a sustained stimulation of the Nuclear Factor kappa-light-chain-enhancer of activated B cells (NF−κB) cascade, which elicits an inflammatory response with the potential to cause cellular demise (Zhou et al., 2022). As the only transmembrane Autophagy-Related Gene (ATG) protein, ATG9A can be palmitoylated by ZDHHC5 at Cys155/156 (Xia et al., 2025). This modification promotes its binding to the Adaptor Protein 4 (AP-4) complex and its trafficking from the Golgi apparatus to the phagophore membrane, thereby ensuring the efficiency of autophagosome initiation and elongation (Xia et al., 2025). Furthermore, research has shown that the S-palmitoylation of Gasdermin D (GSDMD), a crucial pyroptosis protein, is mediated by ZDHHC5/9 (Balasubramanian et al., 2024). This modification directly potentiates the pore-forming capacity of GSDMD. Additionally, the inflammatory mediators liberated during this process can, in turn, indirectly influence autophagy, establishing an intricate network that governs cellular demise (Balasubramanian et al., 2024).

The lysosome has emerged as a vital role in the terminal phase of autophagy, where it fuses with the autophagosome to degrade enclosed contents. Critically, the functional equilibrium and operational state of the lysosome itself are likewise subject to precise regulation via palmitoylation. The palmitoylation-depalmitoylation cycle of lysosomal membrane proteins is essential for maintaining their function. At the level of membrane fusion, the lipid modification of the Soluble N-ethylmaleimide-sensitive factor Attachment protein REceptor (SNARE) protein complex is key to driving the fusion of autophagosomes with lysosomes. In yeast and mammalian cells, the proper membrane localization and function of the R-SNARE protein Synaptobrevin homolog YKT6 (Ykt6) depend on its palmitoylation (Zheng et al., 2024). Crucially, this modification enables Ykt6 to form a priming complex with Syntaxin 17 (STX17) and Synaptosomal-Associated Protein 29 (SNAP29), which facilitates the subsequent recruitment of Vesicle-Associated Membrane Protein 8 (VAMP8) to drive autolysosome formation (Zheng et al., 2024). Interestingly, palmitoylation not only regulates the degradation process but can also conversely protect specific proteins from lysosomal clearance. Studies have shown that the stability of the transcription factor Octamer-binding transcription factor 4A (Oct4A) and its tumorigenicity in Glioblastoma Stem Cells (GSC) depend on ZDHHC17-mediated palmitoylation (Chen et al., 2023). This modification effectively prevents Oct4A from being degraded by the lysosome, thereby maintaining its protein level and function (Chen et al., 2023).

In summary, by regulating nearly all critical steps in the autophagy pathway—from the recruitment of initiation complexes, the elongation of the autophagosome membrane, and the engulfment of selective substrates, to the final fusion with the lysosome—protein palmitoylation constitutes a core regulatory network that determines autophagic efficiency and cell fate.

### 2.3 Necroptosis

As a highly orchestrated version of programmed necrosis, necroptosis is morphologically identified by the cracking of the plasma membrane and subsequent leakage of the cell’s internal components. Mechanistically, however, it depends on a specific signal transduction pathway and is typically activated when the key apoptotic effector, Caspase-8, is functionally inhibited (Pasparakis and Vandenabeele, 2015; Yan et al., 2022). A signaling cascade comprises Receptor-Interacting Protein Kinase 1 (RIPK1), RIPK3, and Mixed Lineage Kinase Domain-like protein (MLKL) constitutes the fundamental mechanism of this process (Hou et al., 2024). If the apoptotic route is suppressed, exposure to triggers such as Tumor Necrosis Factor-alpha (TNFα) results in the activation of RIPK1’s kinase capabilities (Hou et al., 2024). The activated RIPK1 then engages and phosphorylates RIPK3. Together, they assemble into a functional “necrosome”. The downstream effector protein MLKL is then phosphorylated by the active form of RIPK3. This event causes MLKL to oligomerize and relocate to the cell surface. The ultimate loss of membrane integrity and subsequent lytic cell death result from a compromised osmotic balance. This disturbance is caused by pores created in the plasma membrane by assembled MLKL oligomers. (Pasparakis and Vandenabeele, 2015).

The functionality of central proteins and the dictation of cellular fate within this signaling cascade are fundamentally controlled by palmitoylation, an important lipid modification. The efficient assembly of the necrosome is propelled by the S-palmitoylation of RIPK1 (Zhang N. et al., 2024). This modification markedly improves the protein’s trafficking to the cell surface and facilitates its association with the upstream Tumor Necrosis Factor Receptor 1 (TNFR1) complex. (Zhang N. et al., 2024). Critically, when Caspase-8 activity is inhibited, palmitoylated RIPK1 preferentially activates the RIPK3-MLKL axis leading to necroptosis, rather than diverting to the pro-survival NF-κB pathway (Pasparakis and Vandenabeele, 2015). Therefore, the palmitoylation of RIPK1 can be considered a ‘molecular switch’ that determines cell fate, deciding between apoptosis and necroptosis (Pasparakis and Vandenabeele, 2015). Furthermore, regarding the final effector protein MLKL, although the associated mechanisms are still under investigation, preliminary evidence suggests that palmitoylation may play a role after its phosphorylation by RIPK3 (Yan et al., 2022). This modification might further stabilize the active conformation of MLKL and enhance its affinity for plasma membrane phosphatidylinositol phosphates (PIPs), thereby accelerating its oligomerization, pore formation, and the eventual process of cell membrane rupture (Yan et al., 2022).

### 2.4 Pyroptosis

A type of programmed cell death driven by the Gasdermin (GSDM) family and notable for its powerful inflammatory response is known as pyroptosis. The core biochemical event is the cleavage of a GSDM protein by a specific Caspase, which releases the pore-forming N-terminal domain (GSDM-NT) (Shi et al., 2015). Following this cleavage, the GSDM-NT fragment assembles into oligomers at the plasma membrane, creating channels that span the membrane. These pores cause cellular swelling and eventual rupture, along with the liberation of pro-inflammatory mediators like Interleukin-1 beta (IL-1β) and IL-18, which elicits a strong inflammatory reaction (Cookson and Brennan, 2001; Shi et al., 2015). This process is initiated through two main pathways: the canonical pathway, which depends on the activation of Caspase-1; and the non-canonical pathway, which is directly mediated by Caspase-4/5 (human) or Caspase-11 (murine) (Yu et al., 2021; Wu et al., 2022). Recent studies have revealed that dynamic S-palmitoylation, as a key post-translational modification, plays an indispensable role in the activation and execution of pyroptosis by precisely regulating core components of the signaling pathway (Zhang N. et al., 2024). Its main targets include the upstream signal sensor, the NLR family pyrin domain containing 3 (NLRP3) inflammasome, and the downstream core effector, Gasdermin D (GSDMD) (Zhang N. et al., 2024).

The entire signaling lifecycle of the NLRP3 inflammasome, including its initiation, amplification, and termination, is subject to fine-tuned control by palmitoylation events that are specific in time and space. First, during the initial phase of activation, ZDHHC7-mediated palmitoylation maintains NLRP3 in a state prone to liquid-liquid phase separation (Zou et al., 2025). Upon cellular stimulation, this modification enables NLRP3 to rapidly form cytoplasmic puncta and initiate inflammasome assembly (Schmacke and Hornung, 2025; Zou et al., 2025). Conversely, the depalmitoylating enzyme ABHD13 negatively regulates this process (Schmacke and Hornung, 2025; Zou et al., 2025). Second, palmitoylation governs the subcellular localization of NLRP3. For example, palmitoylation catalyzed by ZDHHC1 promotes the dynamic trafficking of NLRP3 between different organelles, ultimately guiding it to the Microtubule-Organizing Center (MTOC), where it interacts with kinases such as NEK7 to achieve full activation (Nie et al., 2024). Additionally, palmitoylation directly enhances interactions within the complex. Palmitoylation at sites Cys837/838 mediated by ZDHHC5, and at site Cys419 mediated by ZDHHC17, respectively enhance the binding of the NLRP3 Leucine-Rich Repeat (LRR) domain to Nima-related kinase 7 (NEK7) and stabilize its NACHT domain (Zheng et al., 2023; Hu et al., 2024). This accelerates inflammasome oligomerization and assembly, processes that can be reversed by depalmitoylating enzymes like ABHD17A (Zheng et al., 2023; Hu et al., 2024). Finally, during the signal termination phase, ZDHHC12 palmitoylates NLRP3 at the Cys844 site, marking it for degradation via the autophagy pathway, whereas PPT1 promotes its degradation through depalmitoylation (Lv et al., 2023; Wang et al., 2023). Thus, dynamic palmitoylation at multiple sites, mediated by multiple enzymes, constitutes a complex network that regulates the lifecycle of the NLRP3 inflammasome.

As the final executor of pyroptosis, the function of GSDMD is directly regulated by palmitoylation at multiple levels, including cleavage, membrane localization, and pore formation. During the activation stage, the palmitoylation of GSDMD at site Cys191 (human) or Cys192 (murine) acts as a critical checkpoint. According to the “relay” model, this modification is catalyzed by ZDHHC7 and serves as a prerequisite for efficient GSDMD cleavage by enhancing the interaction between GSDMD and Caspases (Zhang N. et al., 2024). In the ROS-dependent activation model, other studies have identified ZDHHC5 and ZDHHC9 as the primary acyltransferases responsible for this modification (Balasubramanian et al., 2024; Du et al., 2024). Signals such as ROS can amplify this process specifically by modulating the activity of ZDHHC5/9 or the accessibility of the substrate, linking metabolic stress to GSDMD activation (Du et al., 2024). Notably, the importance of palmitoylation can even surpass the necessity of cleavage; research has shown that cleavage-resistant GSDMD mutants can still form pores when forcibly palmitoylated, whereas a defect in the palmitoylation site completely blocks pyroptosis, even if upstream Caspases have been activated (Du et al., 2024). After cleavage, the palmitoylation of GSDM-NT, mediated by enzymes like ZDHHC5/9 or ZDHHC7, increases its hydrophobicity, driving its targeting and anchoring to cell membranes enriched in lipids such as phosphatidylserine (Balasubramanian et al., 2024; Zhang N. et al., 2024). Subsequently, a divergence in mechanisms exists: the “relay” model specifically proposes a dynamic depalmitoylation step mediated by APT2 to dissociate GSDM-NT from the membrane anchor and facilitate oligomerization (Zhang N. et al., 2024). In contrast, the ROS-dependent activation model suggests that depalmitoylation is not required, and the palmitoylation itself helps to stabilize the pore structure, prolonging the release time of pro-inflammatory factors (Du et al., 2024).

In summary, a dynamic cycle of palmitoylation and depalmitoylation precisely guides the transformation of GSDMD from an inactive precursor protein into an efficient cell death effector molecule. Further investigation is required to reconcile these conflicting models and determine the precise spatiotemporal regulation of GSDMD in diverse pathological contexts. A deeper understanding of this lipid modification switch will not only refine the molecular framework of pyroptosis but also uncover novel pharmacological targets for treating GSDMD-driven inflammatory disorders.

### 2.5 Ferroptosis

Ferroptosis is a type of programmed cell death that is iron-dependent. The core mechanism is the uncontrolled accumulation of intracellular lipid peroxides, which ultimately causes destructive failure of the cell membrane system (Stockwell, 2022). The occurrence of this process depends on an imbalance between pro-oxidative drivers and antioxidant defenses. One part of the process involves ferrous iron (Fe2+) driving the generation of ROS via the Fenton reaction. These ROS then directly target phospholipids containing abundant polyunsaturated fatty acids (PUFAs) within cellular membranes, setting off a damaging cascade of peroxidation (Li et al., 2020). Key lipid metabolism enzymes, including Acyl-CoA Synthetase Long-chain family member 4 (ACSL4) and Lysophosphatidylcholine Acyltransferase 3 (LPCAT3), actively promote the incorporation of PUFAs into phospholipids, thereby providing a rich pool of substrates for peroxidation (Tang et al., 2021). On the other hand, cells have evolved two major defense systems to counteract this process: the canonical Glutathione Peroxidase 4 (GPX4) pathway and the non-canonical Ferroptosis Suppressor Protein 1 (FSP1)-CoQ10 pathway (Jiang et al., 2021; Mishima et al., 2022). Emerging research indicates that palmitoylation, as a key post-translational modification, profoundly influences cellular sensitivity to ferroptosis by regulating core proteins within this defense system.

Palmitoylation constitutes a primary defense mechanism against ferroptosis in the main antioxidant route. This protection is achieved through the stabilization of Solute Carrier Family 7 Member 11 (SLC7A11), which is the vital subunit of the cystine/glutamate antiporter (System Xc^−^). The synthesis of glutathione (GSH), which is essential for the function of GPX4, is limited by the availability of cystine inside the cell. The cellular import of this cystine is the primary responsibility of SLC7A11. Studies have confirmed that the palmitoyltransferase ZDHHC8 can catalyze the palmitoylation of SLC7A11 at its Cys327 site (Wang et al., 2024). This modification greatly enhances the protein stability of SLC7A11, effectively preventing its degradation through the ubiquitin-mediated lysosomal pathway (Wang et al., 2024). Consequently, mutation of this site significantly accelerates SLC7A11 turnover and increases cellular susceptibility to ferroptosis. Furthermore, this regulation is coupled with the cell’s energy status, as AMP-activated protein kinase alpha 1 (AMPKα1) can promote the binding of ZDHHC8 to SLC7A11 through phosphorylation, thereby synergistically enhancing resistance to ferroptosis (Wang et al., 2024).

Beyond regulating the supply of upstream substrates, palmitoylation also exerts direct and precise control over the core effector enzyme of the defense system, GPX4. The function and localization of GPX4, the only selenoprotein capable of directly reducing membrane lipid peroxides, are both influenced by dynamic palmitoylation. Studies have found that the palmitoylation of GPX4 at the Cys66 site enhances its protein stability, which is crucial for maintaining its function in suppressing lipid peroxidation (Huang et al., 2025). In contrast, the acyl-protein thioesterase APT2, identified as the depalmitoylase of GPX4, promotes ferroptosis sensitivity by removing palmitoylation at this site (Huang et al., 2025). This process accelerates GPX4 degradation via the ubiquitin-proteasome pathway, without affecting its localization on the cell membrane. Moreover, recent studies have revealed an even more refined regulatory mechanism: ZDHHC8 can catalyze the palmitoylation of GPX4 at another site, Cys75 (Zhou et al., 2025). This modification specifically guides GPX4 to the endoplasmic reticulum membrane, a site where lipid peroxidation is particularly active (Zhou et al., 2025). This targeted localization allows GPX4 to more efficiently clear nascent lipid peroxides, thereby conferring strong ferroptosis resistance to cells, such as tumor cells. Therefore, palmitoylation at different sites endows GPX4 with the ability to perform its function in different subcellular regions, constituting a complex regulatory network for defending against ferroptosis.

### 2.6 Lysosome-Dependent Death

Lysosome-Dependent Death (LDD), a kind of programmed cell death, is set in motion by the enhanced permeabilization of the lysosomal membrane (LMP). The core event is the disruption of the lysosomal membrane structure, resulting in various hydrolytic enzymes, such as cathepsins, leaking into the cytoplasm (Du et al., 2021). Once these unregulated enzymes enter the cytosol, they cleave key substrates, triggering downstream cascade reactions. For example, Cathepsin B (CTSB) can cleave Bcl-2 family proteins, thereby activating the mitochondria-mediated apoptotic pathway; meanwhile, leaked Zn^2+^ ions may induce mitochondrial swelling and adenosine triphosphate (ATP) depletion, leading to rapid cell necrosis (Du et al., 2021; Liu S. et al., 2024). In fact, LMP is not an isolated event; it often occurs as a convergence point or a consequence of other cell death pathways. For example, the necroptosis effector protein MLKL can directly disrupt the lysosomal membrane, while excessive autophagy can lead to lysosomal overload and rupture (Liu S. et al., 2024; Zhang et al., 2024a). Therefore, LDD is a highly integrated mode of death, closely intertwined with processes such as apoptosis, necroptosis, and autophagy, highlighting the key status of the lysosome as a decision-making center for cell fate (Dai et al., 2024).

Although direct evidence for the palmitoylation-mediated regulation of LMP is still limited, extensive research indicates that this modification can indirectly regulate cellular sensitivity to LDD by affecting proteins related to lysosomal function or upstream pathways. On one hand, palmitoylation can act as a protective mechanism by stabilizing key proteins to prevent their degradation by the lysosome. For example, in glioma, the palmitoylation of the SLC7A11 protein, catalyzed by ZDHHC8, effectively inhibits its ubiquitination and subsequent lysosomal degradation, thereby enhancing the cell’s ability to resist ferroptosis (Wang et al., 2024). Similarly, the palmitoylation of Programmed death-ligand 1 (PD-L1) has also been shown to enhance its protein stability, protecting it from lysosomal clearance (Yao et al., 2019). On the other hand, palmitoylation can also promote upstream processes that may induce LDD. The palmitoylation of the key autophagy protein Autophagy related 16 like 1 (ATG16L1), mediated by ZDHHC7, is necessary for it to efficiently promote autophagosome formation and lysosomal fusion (Wei et al., 2024). Although autophagy is typically a cell survival mechanism, its overactivation can overwhelm the lysosome, potentially leading to LMP and triggering LDD.

Consequently, the choice between using the lysosome for cellular maintenance versus undergoing death from its rupture is indirectly governed by palmitoylation, through its control over the stability and activity of essential proteins.

## 3 Palmitoylation and cell death in disease

### 3.1 The regulatory role of palmitoylation in cancer

**FIGURE 2:**
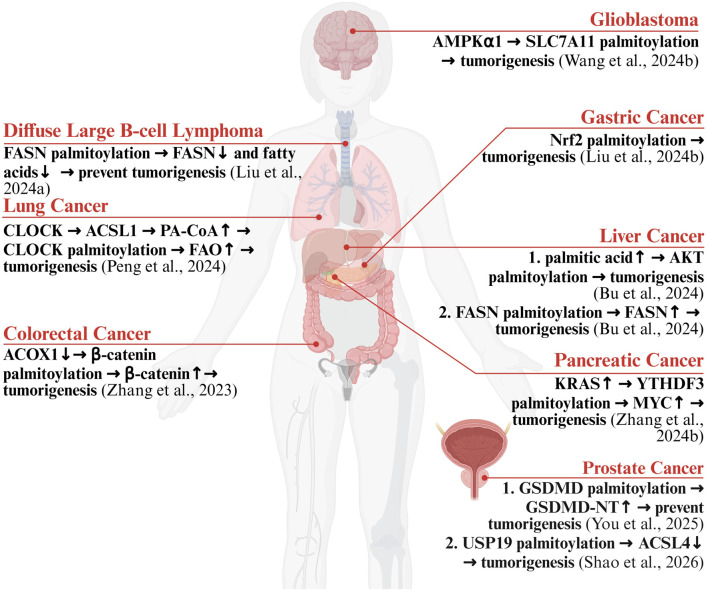
Palmitoylation and Cell Death in Cancer. FASN: Fatty Acid Synthase; CLOCK: Circadian Locomotor Output Cycles Kaput; ACSL: Acyl-CoA Synthetase Long-chain family member; PA-CoA: palmitoyl-CoA; FAO: Fatty Acid Oxidation; ACOX1: Acyl-CoA Oxidase 1; Nrf2: Nuclear factor rythroid 2-related factor 2; PKB/AKT: Protein Kinase B; KRAS: Kirsten Rat Sarcoma viral oncogene homolog; YTHDF3 YTH domain family member 3; MYC Myelocytomatosis viral oncogene homolog; GSDMD Gasdermin D; NT N-terminal domain; AMPKα1: AMP-activated protein kinase alpha 1; SLC7A11: Solute Carrier Family 7 Member 11; USP19: Ubiquitin-Specific Peptidase 19.

In the context of oncology, S-palmitoylation has multifaceted and context-specific functions. Through its dynamic control over the stability, spatial distribution, and activity of pivotal proteins, this acylation process significantly affects cancer initiation, metabolic shifts, metastasis, and immune surveillance. Notably, the function of palmitoylation varies significantly across different cancer types, acting as either a carcinogenic driver or a tumor suppressor, making it a therapeutic target fraught with both challenges and opportunities (Figure 2).

In many malignancies, aberrant palmitoylation promotes disease progression by stabilizing key oncoproteins or activating oncogenic signaling pathways. To illustrate, when the tumor suppressor Acyl-CoA Oxidase 1 (ACOX1) is downregulated in colorectal cancer, palmitic acid (PA) accumulates (Zhang et al., 2023). This accumulated PA subsequently encourages the palmitoylation and stabilization of β-catenin, which finally results in the uncontrolled activation of the Wnt/β-catenin pathway (Zhang et al., 2023). A high-fat diet provides a source of palmitic acid that, in liver cancer, can be used by ZDHHC17/24 to drive the S-palmitoylation of Protein Kinase B (AKT, also known as PKB) (Bu et al., 2024). This allows AKT to anchor to the cell membrane and be activated independent of the canonical Phosphatidylinositol (3,4,5)-trisphosphate (PIP3) pathway, thereby driving liver cancer development (Bu et al., 2024). Furthermore, palmitoylation is involved in complex signaling feedback loops. In specific tumor models like sleep deprivation-induced lung cancer, the main regulator of circadian rhythms, Circadian Locomotor Output Cycles Kaput (CLOCK) becomes transcriptionally hyper-activated (Peng et al., 2024). This heightened activity of CLOCK then enhances the expression of Acyl-CoA Synthetase Long-chain family member 1 (ACSL1) (Peng et al., 2024). The elevated ACSL1 subsequently facilitates the production of palmitoyl-CoA (PA-CoA). PA-CoA, catalyzed by ZDHHC5, then mediates the palmitoylation of CLOCK itself (Peng et al., 2024). This modification inhibits the ubiquitination-mediated degradation of CLOCK, thus forming a positive feedback loop. The stabilized CLOCK protein continuously activates the Fatty Acid Oxidation (FAO) pathway, enhancing cancer stem cell properties associated with sleep deprivation (Peng et al., 2024). In addition to directly regulating signaling molecules, palmitoylation is also essential for the metabolic reprogramming of tumors, primarily by stabilizing key enzymes and regulatory factors in metabolic pathways. Fatty Acid Synthase (FASN) is a key anabolic enzyme that is hyperactive in many tumor cells. In liver cancer, ZDHHC20-mediated palmitoylation of FASN protects it from proteasomal degradation, thereby stabilizing FASN protein levels and providing ample lipid raw materials for tumor growth (Bu et al., 2024). The malignant characteristics of pancreatic tumors can be promoted by the oncoprotein Myelocytomatosis viral oncogene homolog (MYC) (Zhang et al., 2024b). Its abnormal accumulation is caused by the stabilization of its regulatory protein, YTH domain family member 3 (YTHDF3). This stabilization results from YTHDF3’s S-palmitoylation, which inhibits its degradation. This entire process is initiated by high levels of the ZDHHC20 enzyme, which is expressed due to mutations in the Kirsten Rat Sarcoma viral oncogene homolog (KRAS) oncogene (Zhang et al., 2024b). The progression of gastric tumors is also driven by another key event: the S-palmitoylation of Nuclear factor erythroid 2-related factor 2 (Nrf2), which is mediated by the enzyme ZDHHC2 (Liu L. et al., 2024). In glioblastoma, the S-palmitoylation of SLC7A11 serves as a critical mechanism for suppressing ferroptosis and sustaining malignant tumor growth (Wang et al., 2024). This post-translational modification is catalyzed by the ZDHHC8 at Cys327, which stabilizes the SLC7A11 protein by attenuating its ubiquitination and preventing lysosome-mediated degradation (Wang et al., 2024). Mechanistically, the upstream kinase AMPKα1 directly phosphorylates ZDHHC8 at Ser299; this phosphorylation enhances the interaction between ZDHHC8 and SLC7A11, thereby facilitating SLC7A11 S-palmitoylation (Wang et al., 2024). Consequently, this AMPKα1-ZDHHC8-SLC7A11 regulatory axis confers ferroptosis resistance upon tumor cells and correlates with poor patient prognosis. In a similar vein, ZDHHC2-mediated S-palmitoylation has been identified as a key driver of ferroptosis resistance and enzalutamide tolerance in castration-resistant prostate cancer (CRPC) (Shao et al., 2026). Specifically, upregulated ZDHHC2 catalyzes the S-palmitoylation of the deubiquitinase Ubiquitin-Specific Peptidase 19 (USP19) (Shao et al., 2026). This modification disrupts the interaction between USP19 and its substrate ACSL4, thereby abrogating the deubiquitination-mediated stabilization of ACSL4 and promoting its degradation (Shao et al., 2026). As ACSL4 acts as a key executioner of ferroptosis, its downregulation suppresses the accumulation of lethal lipid peroxides, ultimately conferring a survival advantage upon tumor cells.

In stark contrast to the pro-tumorigenic functions described above, however, palmitoylation can also exert a tumor-suppressive role in certain contexts or become a therapeutic target for inducing cancer cell death. A typical example also involves the regulation of FASN. Reduced levels of the palmitoyltransferase ZDHHC21 are a notable feature of Diffuse Large B-cell Lymphoma (DLBCL) (Liu B. et al., 2024). Its normal function is to catalyze the palmitoylation of FASN at the Cys1317 site (Liu B. et al., 2024). However, modification at this specific site paradoxically decreases the stability of FASN, thereby inhibiting fatty acid synthesis and cancer cell growth. Therefore, reduced ZDHHC21 expression levels correlate with unfavorable clinical outcomes for individuals with DLBCL. (Liu B. et al., 2024). This finding reveals that different enzymes and modification sites can produce diametrically opposed functions when regulating the same protein. Furthermore, targeting palmitoylation to drive programmed cell death has also shown therapeutic potential. In a prostate cancer model, the combination of Astragaloside IV and scorpion venom polypeptide was found to boost ZDHHC1 expression (You et al., 2025). This, in turn, improved the S-palmitoylation of the GSDMD N-terminus and its subsequent recruitment to the cell surface (You et al., 2025). This ultimately led to the effective promotion of pyroptosis in cancer cells, slowing tumor progression.

### 3.2 The role of palmitoylation in neurodegenerative diseases

**FIGURE 3:**
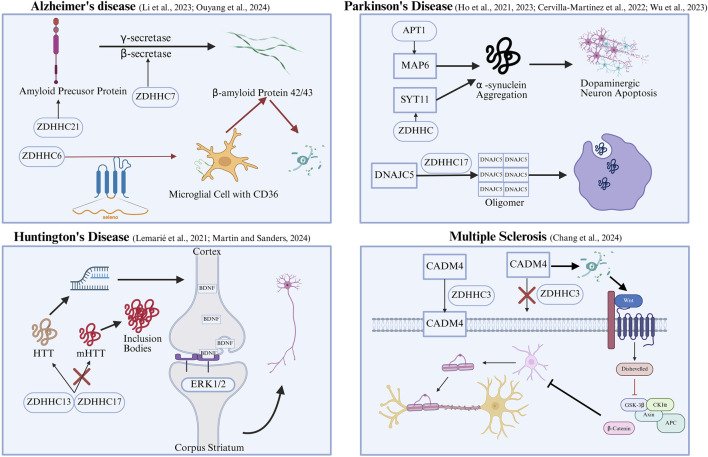
Palmitoylation and Cell Death in Neurodegenerative Diseases. ZDHHC: Zinc finger DHHC-type palmitoyltransferase; CD36: Cluster of Differentiation 36; MAP6: Microtubule-associated protein 6; Syt11: Synaptotagmin-11; CSPα/DNAJC5: Cysteine String Protein alpha; mHTT: mutant Huntingtin protein; BDNF: Brain-Derived Neurotrophic Factor; ERK1/2: Extracellular signal-regulated kinase 1 and 2; Cadm4: Cell adhesion molecule 4.

Within the nervous system, S-palmitoylation is vital for the proper development of neurons, the plasticity of synapses, and cellular signaling. As a result, when this dynamic process is dysregulated, it is connected to the pathology of a range of neurodegenerative disorders. Faulty palmitoylation can substantially hasten the advancement of these conditions by affecting cellular demise pathways like apoptosis, pyroptosis, and programmed necrosis (Figure 3).

#### 3.2.1 Alzheimer’s disease

In Alzheimer’s Disease (AD), aberrant palmitoylation is comprehensively involved in the core pathological processes of the disease by affecting the generation and elimination of amyloid-beta (Aβ), as well as its downstream toxic effects. First, regarding Aβ generation, the palmitoylation of both Amyloid Precursor Protein (APP) and its processing enzymes, including β-secretase, promotes their interaction and cleavage efficiency at the membrane (Bhattacharyya et al., 2013; Cho and Park, 2016). In particular, ZDHHC21-mediated palmitoylation of APP has been shown to direct it toward an aberrant cleavage pathway, producing more aggregation-prone and neurotoxic Aβ42/43 fragments (Li et al., 2023). Second, regarding Aβ clearance, the phagocytic function of microglia is also regulated by palmitoylation. Impaired clearance of amyloid-beta (Aβ), which exacerbates neuroinflammation and harms neurons, can result from defective palmitoylation of the CD36 receptor (Ouyang et al., 2024). This crucial modification, facilitated by the Selenoprotein K (SELENOK)/ZDHHC6 axis, is normally required for the receptor’s correct localization to the cell surface and for efficient Aβ removal. (Ouyang et al., 2024). Furthermore, palmitoylation mediates the downstream neurotoxicity of Aβ. In AD models, elevated levels of palmitoylation on the postsynaptic scaffolding protein PSD-95 lead to its abnormal localization and overactivation of the N-methyl-D-aspartate receptor subtype 2B (NMDAR2B) receptor, triggering excessive calcium influx and excitotoxicity, which ultimately results in neuronal death (Zhang et al., 2022).

#### 3.2.2 Parkinson’s disease

In Parkinson’s Disease (PD), the protein palmitoylation profile of the entire brain is significantly altered, and the pathogenic process of the key pathogenic protein, alpha-synuclein (α-syn), is subject to complex and multifaceted regulation by palmitoylation. This regulation exhibits a dual nature: on one hand, certain modifications have a protective effect, such as the palmitoylation of the Microtubule-associated protein 6 (MAP6) protein, which can reduce the phosphorylation level and cytotoxicity of α-syn. On the other hand, other modifications can exacerbate pathological damage. For example, the palmitoylation of Synaptotagmin-11 (Syt11) enhances the binding of α-syn to intracellular membranes and promotes its aggregation, ultimately inducing the apoptosis of dopaminergic neurons (Ho et al., 2021; 2023; Cervilla-Martínez et al., 2022). The process of clearing α-syn is another biological function critically influenced by palmitoylation. The palmitoylation of the Cysteine String Protein alpha (CSPα/DNAJC5) protein is necessary for its membrane anchoring and for mediating the secretion of α-syn out of the cell via a non-canonical pathway (Wu et al., 2023). If this palmitoylation process is inhibited, the extracellular secretion of α-syn is blocked, leading to its intracellular accumulation and exacerbating its toxic effects (Wu et al., 2023).

#### 3.2.3 Huntington’s disease

A deficient S-palmitoylation state of the mutant Huntingtin protein (mHTT) is tightly connected to its toxic gain-of-function. This toxicity is the fundamental driver of the core pathology seen in Huntington’s Disease (HD). The enzymes ZDHHC17 and ZDHHC13 efficiently catalyze the S-palmitoylation of the normal HTT protein at its Cys214 residue (Lemarié et al., 2021). This acylation is vital for proper membrane localization, subcellular trafficking, and the protein’s role in supporting the transport of Brain-Derived Neurotrophic Factor (BDNF). (Yanai et al., 2006; Saudou and Humbert, 2016; Lemarié et al., 2021; Kacher et al., 2022). However, in mHTT, the level of palmitoylation at this critical site is significantly reduced. This “hypo-palmitoylated” state leads to an abnormal metabolic pathway for mHTT, making it more prone to intracellular misfolding and the formation of toxic aggregates and inclusion bodies. Experimental evidence shows that further preventing palmitoylation at the Cys214 site through genetic mutation significantly exacerbates the HD pathological phenotype in neuronal cultures (Martin and Sanders, 2024). Therefore, the reduced palmitoylation of mHTT is a key upstream event leading to its neurotoxicity, ultimately driving disease progression by inducing neuronal apoptosis.

#### 3.2.4 Multiple Sclerosis

Multiple Sclerosis (MS) is an autoimmune condition notable for the loss of myelin sheaths within the Central Nervous System (CNS). In this disease, abnormal S-palmitoylation has a direct impact on the process of remyelination. The core of this pathological mechanism involves oligodendrocytes, the cells responsible for myelination. Studies have showed that the palmitoylation of the cell adhesion protein Cell adhesion molecule 4 (Cadm4), catalyzed by ZDHHC3, is crucial for maintaining its stability and function on the oligodendrocyte membrane (Chang et al., 2024). A defect in Cadm4 palmitoylation causes it to detach from the cell membrane and undergo accelerated degradation. This, in turn, results in the inappropriate stimulation of the WNT/β-Catenin signaling pathway, which inhibits oligodendrocyte differentiation and myelin regeneration. In animal models, mice with defective Cadm4 palmitoylation exhibit typical MS pathological features, including abnormal myelin development, cognitive decline, and exacerbated neuroinflammation (Chang et al., 2024). These findings suggest that targeting the ZDHHC3-Cadm4 palmitoylation axis could be an effective therapeutic strategy for promoting myelin repair and ameliorating the inflammatory state in MS (Chang et al., 2024).

### 3.3 Regulation by palmitoylation in immune and inflammatory diseases

S-palmitoylation plays a crucial role in regulating the function of immune cells and inflammatory signaling cascades. This transient lipidation is fundamental to both preserving immune balance and promoting disease-related inflammation through its modulation of the stability, spatial distribution, and activity of essential immune receptors, transcription factors, and signaling hubs. Consequently, targeting the enzymes involved in this process offers a novel therapeutic approach for treating autoimmune disorders and associated inflammatory conditions.

Protein palmitoylation is a critical mechanism for controlling the lifecycle of essential proteins in innate and adaptive immunity. A clear illustration of this is seen in Systemic Lupus Erythematosus (SLE), where a tightly regulated cycle of palmitoylation and its reversal governs the function of Toll-like Receptor 9 (TLR9). Palmitoylation catalyzed by DHHC3 is accountable for trafficking TLR9 from the Golgi apparatus to the endosome, while subsequent depalmitoylation mediated by PPT1 controls its activation within the endosome. Inhibiting either step of this cycle can reduce TLR9 signaling, thereby decreasing the production of type I interferon-alpha (IFN-α). Based on this mechanism, PPT1 inhibitors have shown potential in alleviating nephritis symptoms in SLE patients (Ni et al., 2024). In the context of immunosuppression, the stability of Forkhead box P3 (Foxp3), the core transcription factor for Regulatory T cells (Tregs), depends on cooperative palmitoylation by multiple DHHC enzymes, notably DHHC2. This lipid modification preserves the suppressive function of Tregs. However, the absence of DHHC2 reduces Foxp3 protein levels, compromises this suppressive capacity, and consequently strengthens the anti-tumor immune response (Zhou et al., 2024). In antiviral innate immunity, Mitochondrial Antiviral-Signaling protein (MAVS) is palmitoylated by ZDHHC7 following viral infection. Since this modification is vital for stabilizing mitochondrial oligomers and for effectively transducing antiviral signals, ZDHHC7 is positioned as a possible therapeutic target for autoimmune diseases related to MAVS (Liu Y. et al., 2024).

The NLRP3 inflammasome acts as a critical nexus for pyroptosis and the resulting inflammatory reactions. Therefore, its activation pathway offers an excellent model for how aiming at palmitoylation could be used to treat inflammatory disorders. Aberrant activation of NLRP3 is closely associated with numerous inflammatory diseases. Studies have confirmed that a critical step in its activation is the palmitoylation at the Cys126 site, catalyzed by ZDHHC7. This modification is necessary for the proper localization of NLRP3 to the Trans-Golgi Network (TGN) and the recruitment of the downstream adaptor protein Apoptosis-associated speck-like protein containing a CARD (ASC), thereby driving the assembly of the complete inflammasome. Inhibiting this step can effectively mitigate inflammatory responses such as endotoxic shock (Yu et al., 2024; Zou et al., 2025). In a murine model of sepsis, ZDHHC5 deficiency or its pharmacological inhibition via 2-BP reduced serum levels of IL-1β and IL-18, thereby significantly improving survival rates. These findings suggest that blocking ZDHHC5-mediated NLRP3 palmitoylation is sufficient to abrogate the initiation of the systemic cytokine storm (Yu et al., 2024). Crucially, this regulatory mechanism has direct clinical translational value. One of the mechanisms of action for Disulfiram, a drug approved by the FDA, is to specifically block the palmitoylation of NLRP3, thereby inhibiting inflammasome activation (Zou et al., 2025). This provides a safe and effective clinical therapeutic candidate for NLRP3-related inflammatory diseases.

## 4 Conclusion and perspectives

As a transient and reversible post-translational modification, protein S-palmitoylation fundamentally governs the equilibrium between cellular survival and demise. The specific regulatory functions of palmitoylation within multiple pathways of programmed cell death have been comprehensively detailed throughout this review. These pathways encompass apoptosis, autophagy-dependent cell death, necroptosis, pyroptosis, ferroptosis, and lysosome-dependent cell death. This lipid attachment, which involves the covalent linkage of palmitate, provides fine-tuned control over the stability, spatial distribution, and binding partnerships of essential proteins, thus deciding the cell’s final outcome. This lipid modification is of fundamental importance across these varied pathways of cellular demise. For instance, it governs the localization of Bax to the mitochondria during apoptosis and facilitates the recruitment of RIPK1 to the membrane in necroptosis. Furthermore, it directs the formation of the NLRP3 inflammasome and GSDMD’s pore-forming ability in pyroptosis, while also upholding the function of the SLC7A11/GPX4 axis to defend against ferroptosis. Furthermore, abnormal regulation of this process has been associated with the disease mechanisms of major human ailments, including tumours, neurodegenerative diseases and immune-inflammatory conditions. This robust correlation highlights its noteworthy potential as a promising target for upcoming treatments.

The elucidation of these molecular mechanisms provides highly attractive new targets and strategies for disease intervention. In the field of oncology, new therapeutic avenues have opened by using pharmaceuticals to modulate protein palmitoylation. These strategies include targeting the palmitoylation of specific oncoproteins, such as FASN and β-catenin, and inducing forms of palmitoylation-dependent cell death, including pyroptosis (You et al., 2025). In neuroscience, correcting the aberrant palmitoylation levels in neurodegenerative diseases—for instance, by enhancing the modification of mHTT in Huntington’s disease or modulating the processing of APP in Alzheimer’s disease—may be an effective means of slowing disease progression (Yanai et al., 2006; Bhattacharyya et al., 2013). In immunology, targeting palmitoylation has already achieved clinical translation; the FDA-approved drug Disulfiram controls inflammation by inhibiting NLRP3 palmitoylation. This provides a strong rationale and a successful paradigm for developing more small-molecule modulators targeting specific DHHC enzymes or depalmitoylating enzymes (Zou et al., 2025).

Despite significant progress, the field still faces numerous challenges and is ripe with opportunities. First, the substrate specificities of the 23 DHHC enzymes and various depalmitoylating enzymes in the human genome, as well as their functional division of labor in specific pathological processes, remain to be systematically characterized (Fan et al., 2024). Another crucial direction for future studies involves the dynamic interplay between palmitoylation and other PTMs, including phosphorylation and ubiquitination. This includes understanding the specific mechanisms by which these modifications jointly control cellular signaling (Venne et al., 2014). Finally, developing highly potent and specific small-molecule drugs targeting particular palmitoylating or depalmitoylating enzymes is key to achieving precision therapy and minimizing off-target effects (Abdulrahman et al., 2025).

In conclusion, protein palmitoylation is a critical molecular switch that determines the life-or-death fate of a cell. In-depth analysis of its complex regulatory network not only greatly enriches our understanding of cell death as a fundamental biological process but also provides a solid theoretical foundation for developing innovative therapies that target cell death pathways. These studies hold considerable promise for delivering revolutionary new treatments for major conditions in the coming years.
